# Parenting in a warming world: thermoregulatory responses to heat stress in an endangered seabird

**DOI:** 10.1093/conphys/coz109

**Published:** 2020-01-18

**Authors:** Timothée R Cook, Rowan Martin, Jennifer Roberts, Henry Häkkinen, Philna Botha, Corlia Meyer, Emilee Sparks, Leslie G Underhill, Peter G Ryan, Richard B Sherley

**Affiliations:** 1 FitzPatrick Institute of African Ornithology, DST-NRF Centre of Excellence, University of Cape Town, Rondebosch 7701, South Africa; 2 BLOOM Association, 62 Bis Avenue Parmentier, 75011 Paris, France; 3 Centre for Ecology and Conservation, University of Exeter, Penryn, TR10 9FE UK; 4 Animal Demography Unit, Department of Biological Sciences, University of Cape Town, Rondebosch 7701, South Africa; 5 Centre for Research on Evaluation, Science and Technology, Stellenbosch University, Stellenbosch 7600, South Africa; 6 Environment and Sustainability Institute, University of Exeter, Penryn TR10 9FE, UK

**Keywords:** Breeding, dehydration, evaporative cooling, heat stress physiology, thermoregulation, videography

## Abstract

The frequency of extreme weather events, including heat waves, is increasing with climate change. The thermoregulatory demands resulting from hotter weather can have catastrophic impacts on animals, leading to mass mortalities. Although less dramatic, animals also experience physiological costs below, but approaching, critical temperature thresholds. These costs may be particularly constraining during reproduction, when parents must balance thermoregulation against breeding activities. Such challenges should be acute among seabirds, which often nest in locations exposed to high solar radiation and predation risk. The globally endangered bank cormorant *Phalacrocorax neglectus* breeds in southern Africa in the winter, giving little scope for poleward or phenological shifts in the face of increasing temperatures. Physiological studies of endangered species sensitive to human disturbance, like the bank cormorant, are challenging, because individuals cannot be captured for experimental research. Using a novel, non-invasive, videographic approach, we investigated the thermoregulatory responses of this seabird across a range of environmental temperatures at three nesting colonies. The time birds spent gular fluttering, a behaviour enhancing evaporative heat loss, increased with temperature. Crouching or standing birds spent considerably less time gular fluttering than birds sitting on nests (*ca* 30% less at 22°C), showing that postural adjustments mediate exposure to heat stress and enhance water conservation. Crouching or standing, however, increases the vulnerability of eggs and chicks to suboptimal temperatures and/or expose nest contents to predation, suggesting that parents may trade-off thermoregulatory demands against offspring survival. We modelled thermoregulatory responses under future climate scenarios and found that nest-bound bank cormorants will gular flutter almost continuously for several hours a day by 2100. The associated increase in water loss may lead to dehydration, forcing birds to prioritize survival over breeding, a trade-off that would ultimately deteriorate the conservation status of this species.

## Introduction

The effects of climate change on animals have been a focus of research for over four decades (Parmesan, 2006; Urban, 2015). Dramatic alterations in species’ distributions and abundances have been linked to rising temperatures (Spooner *et al.*, 2018). Indirect, biotic mechanisms (Ockendon *et al.*, 2014), such as changes to species’ phenology and distributions in response to temporal or spatial shifts of resources, are recognized as important mediators of climatic impact on animal populations (e.g. Chen *et al.*, 2011; Ovaskainen *et al.*, 2013). However, direct, abiotic mechanisms may also play a decisive role. In particular, the direct effects of increasing thermoregulatory demands on the animals themselves may affect the development, reproduction, dispersal and survival of ectothermic species (Angilletta, 2009).

Among endotherms (here defined as birds and mammals), thermal homeostasis is often assumed to be disconnected from environmental conditions (Berteaux *et al.*, 2006; La Sorte and Jetz, 2010; Oswald and Arnold, 2012), but there is growing evidence that ‘heat stress’ during hot weather events can directly impact animals by forcing trade-offs between thermoregulatory demands and investment in other physiological functions (e.g. Porter and Kearney, 2009; Smit *et al.*, 2016; Mitchell *et al.*, 2018). These trade-offs can be mediated through both physiological and behavioural processes. For example, animals may have to expend energy and water on dissipating heat at the cost of investing in growth or reproduction, or they may have to adjust their behaviour to exploit cooler environments at the costs of foraging efficiency, territorial defence and parental care (e.g. du Plessis *et al.*, 2012; Cunningham *et al.*, 2013; Woodroffe *et al.*, 2017). Understanding the way in which these trade-offs are resolved will help determine how species respond to rising temperatures.

Such trade-offs can be particularly acute during breeding: managing additional heat loads can have significant impacts on parental behaviour and, in turn, breeding success (e.g. Cunningham *et al.*, 2013; Woodroffe *et al.*, 2017). These impacts are not only limited to animals living in hot, arid environments, but have also been recorded in relatively cool environments (Campagna and Le Boeuf, 1988; Gaston *et al.*, 2002; Oswald *et al.*, 2008), suggesting that the direct effects of rising temperatures might be more widespread than previously recognized. Seabirds, in particular, may be expected to encounter significant thermoregulatory challenges during breeding. Seabirds often breed in exposed locations, such as cliffs or rocky promontories with little protection from the sun, wind and rain, leading to rapid exchanges of heat (both gain and loss) between individuals and their environment. As a result, nesting seabirds often experience environmental temperatures far in excess of local air temperatures (*T*_air_) while attending to eggs and offspring (Schreiber and Burger, 2002). At the same time, seabirds need efficient insulation to limit heat loss while foraging in the ocean (particularly diving species). Thus, adaptations to limit heat loss during foraging, or rapidly gain heat following dives, may be in direct evolutionary conflict with adaptations to dissipate excess heat or avoid heat gain while attending nest sites (Oswald and Arnold, 2012).

Instances of nest abandonment in seabird colonies (e.g. Gaston *et al.*, 2002; Oswald *et al.*, 2008; Sherley *et al.*, 2012) or reductions in adult survival (Ganendran *et al.*, 2016) have been observed during heat waves, suggesting that some seabird populations already experience hyperthermia or dehydration when faced to temperatures exceeding critical thresholds. Although less obvious, temperatures below, but approaching such thresholds, may negatively affect both adults and offspring through increasing demands for body water for evaporative heat loss, or reducing the ability of parents to protect nests from predators (Oswald *et al.*, 2008). Understanding how parents adjust their behaviour during hot weather events may help to identify biologically meaningful temperature thresholds, thereby improving the realism of predictions of climate change impacts on avian communities (Albright *et al.*, 2017; Conradie *et al.*, 2019).

We examined the thermoregulatory responses to high temperatures in breeding bank cormorants *Phalacrocorax neglectus*. The bank cormorant is a range-restricted species confined to the Benguela upwelling region and is listed as globally endangered (Cook, 2015; Roux and Kemper, 2015) due to rapid declines in populations, largely considered to result from a reduction in their prey-base and competition with lobster fisheries (Crawford *et al.*, 1999; Sherley *et al.*, 2017). However, hot weather events have been occurring with increasing frequency across the species’ breeding range in coastal southern Africa (Kruger and Sekele, 2013) and bank cormorants have been observed to abandon nests during heat waves (Sherley *et al.*, 2012), suggesting that rising temperatures may be exacerbating their population decline. Breeding bank cormorants are likely to be particularly susceptible to hot weather events because they have limited scope to adjust by shifting breeding in time (they are winter breeders) or space (they are confined to the southern tip of Africa) (Hockey *et al.*, 2005).

Because of its unfavourable conservation status, its small numbers (less than 2600 breeding pairs globally in 2015; Cook, 2015) and its high sensitivity to human disturbance (approaching a colony may trigger stampedes and collective nest abandonments leading to nest content depredation by aerial predators; Hockey *et al.*, 2005), the bank cormorant cannot be captured for the study of its physiology. We therefore use a novel videographic approach to monitor the thermoregulatory responses of the bank cormorant non-invasively. Digital photography and videography are already being used to monitor wildlife non-intrusively, including studying population dynamics (Sherley *et al.*, 2010), sampling cryptic or elusive species (O’Connell *et al.*, 2011), quantifying activity budgets (Lynch *et al.*, 2015), estimating breeding success (Merkel *et al.*, 2016), describing animal diet (Gaglio *et al.*, 2017) and counting individuals (Hodgson *et al.*, 2018). Surprisingly, only two studies (AlRashidi, 2016; Clauser and McRae, 2017) report the use of these technologies to monitor thermoregulatory responses in wild animals and only in birds (excluding studies using thermal imaging or infrared cameras, which focus on body temperature rather than behaviour; McCafferty, 2013).

By filming birds from a distance over long periods of time, we were able to investigate non-invasively patterns of gular fluttering in wild bank cormorants, a behaviour that enhances heat dissipation through evaporative heat loss but may ultimately lead to dehydration when sustained. We examined how this behaviour relates to parental behaviour during nest attendance in relation to changes in temperature, wind speed and humidity. This approach allows us to predict how patterns of behaviour will change under various climate change scenarios and to explore the potential impacts of rising temperatures for southern African seabirds and for seabirds in general.

## Materials and Methods

### Research permits

Fieldwork was carried out with permission from the Department of Environmental Affairs (RES2012/25 and RES2012/83), CapeNature (AAA007-00013-0056 and 0035-AAA004-000709) and South African National Parks (permit issued on 18 May 2012). The study was approved by the University of Cape Town’s Science Faculty Animal Ethics committee (R2012/2009/V8/TC).

### Study model and sites

The bank cormorant is a large (*ca* 2 kg) marine-obligate cormorant that specializes on inshore benthic prey captured by diving within *ca* 10 km of the colony (Cooper, 1985; Ludynia *et al.*, 2010). We studied adult bank cormorants on their nests during their breeding season (April to August) in 2012. Field work was conducted at three breeding colonies near Cape Town, in South Africa’s Western Cape Province (Fig. S1). The Jutten Island colony (33°05′S, 17°57′E) is situated on top of large granite boulders near the water line, the Robben Island colony (33°48′S, 18°22′E) is located on top of a short harbour wall comprising interlocking cement blocks (‘dolosse’) and the Stony Point colony (34°22′S, 18°53′E) is a mainland colony perched upon a large granite outcrop close to the high-tide line. The breeding populations at Jutten Island, Robben Island and Stony Point held 19, 93 and 32 breeding pairs, respectively, in 2012 (Sherley *et al.*, 2017). Due to the modest size of these colonies, data collected at different localities were combined in all subsequent analyses (see below).

### Recording air (*T*_air_) and operative (*T*_bb_) temperature

The thermal environment that an organism experiences at any point in time and space is determined by the physical properties of the organism as well as prevailing climatic parameters including air temperature (*T*_air_), wind speed and solar radiation. Thus, *T*_air_, recorded sheltered from precipitation and direct sunlight—sometimes called ambient temperature—can differ significantly from the thermal environment an organism experiences in the field. Operative temperature, which corresponds to the intuitive concept of the temperature of the environment (Bakken *et al.*, 1985), measures ‘the thermal environment on a scale relevant to an animal’s microhabitat, by integrating convective and radiative heat transfer between the environment and the animal’ (Dzialowski, 2005; p. 1). Here, the thermometer is a test object with similar external properties to the animal, and the thermometric property is net heat flow to/from the test object (Bakken, 1992). Thus, operative temperature helps understand whether an organism is gaining or losing heat depending on the organism’s properties (size, shape or posture) and those of the environment (temperature, solar radiation, humidity, wind; Bakken and Angilletta, 2014). Operative temperature thermometers have been used broadly across taxa to study the relationship between an animal’s thermal environment and its physiology and ecology (Angilletta, 2009). However, such an approach has two key limitations: first, it is extremely challenging to produce a physical model that replicates the physical properties of an organism (Bakken, 1992) and second, it is rarely possible to place thermometers in locations that precisely mimic the microclimatic conditions experienced by the study organism, particularly if that organism is a threatened species sensitive to human disturbance. Therefore, operative temperature thermometers generally only provide an approximation of true operative temperature (Bakken and Angilletta, 2014). We constructed an operative temperature thermometer based on a ‘black bulb’, using a painted metal sphere sized to approximate the dimensions of a bank cormorant, following Walsberg and Weathers (1986). These black bulbs were placed in the field in sites that reflected the thermal surroundings of the animals under the study conditions, providing a relative measure of the thermal environment of study birds (*T*_bb_) that is not approximated by *T*_air_ alone (Cunningham et al., 2015).

**Figure 1:**
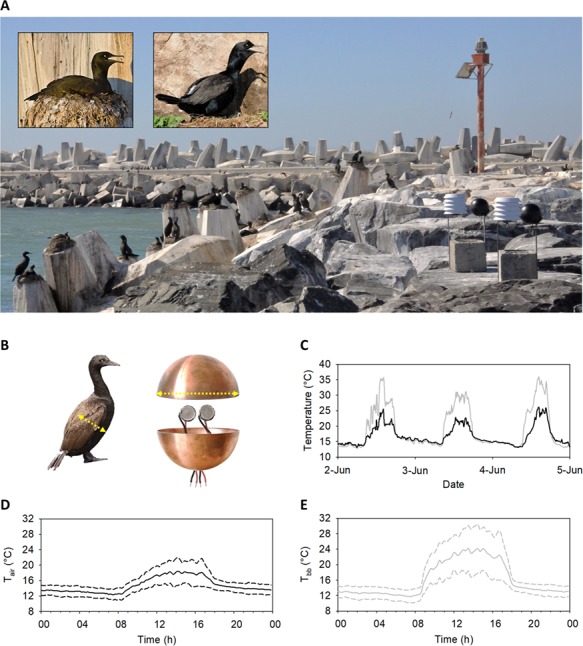
Field set-up for monitoring bird behaviour in relation to temperature. (A) View of the Robben Island bank cormorant colony (photo: Timothée Cook). Cormorant study nests are visible in the background. Here, video recorders are set up 20 m from the first nests (the photo represents a subjective view of the colony as seen through the cameras). Stevenson screens and spheres recording respectively air (*T*_air_) and black bulb (*T*_bb_) temperatures are visible in the right foreground. The inset photos show examples of two typical postures recorded in nesting cormorants (here seen gular fluttering): sitting (left, photo: Davide Gaglio) and crouching (right, photo: Johannes Pfleiderer). (B) Inside a copper sphere, with temperature loggers visible. Sphere diameter was equal to the dorso-ventral distance (11.5 cm) measured in a bank cormorant (shown on a standing bird, photo: Mike Buckham). (C) Example of *T*_air_ (black line) and *T*_bb_ (grey line) recorded over 3 days in 2012. (D) Example of mean *T*_air_ over the daily cycle (solid line) ± SD (dashed line) recorded over a 2-week period (30 May 2012 to 12 June 2012). (E) Example of mean *T*_bb_ over the daily cycle (solid line) ± SD (dashed line) recorded over the same period.

We recorded *T*_air_ and *T*_bb_ using iButton loggers (model: D51922L-F5#) from Maxim (San Jose, CA, USA), accurate to 0.065°C for a range of −40 to 85°C. We used ColdChain Thermodynamics software (Fairbridge Technologies, Sandton, South Africa) for iButton programming and temperature data extraction. Prior to use, we calibrated the iButtons using water baths, discarding those outside a 0.3°C temperature range. *T*_air_ inside the colony was recorded with conventional white, cylindrical Stevenson screens measuring 13 cm in diameter and 15 cm in height (Fig. 1A). We inserted two iButtons into each screen, in custom made iButton holders. *T*_bb_ was recorded using black copper spherical models designed to approximate the thermal properties of adult bank cormorants, following Walsberg and Weathers (1986). Each model was assembled from hollow copper alloy hemispheres, 11.5 cm in diameter and 0.8 mm thick. The diameter for these spheres was calculated by measuring the chest circumference (just behind the wings) of a dead bank cormorant (Fig. 1B). Two iButton holders were inserted through a hole in the lower half-sphere so that the iButtons were held near the centre of the sphere, recording *T*_air_ inside (Fig. 1B). We then bolted, superglued and taped the two hemispheres together with Tesa Tape (No. 4651; Beiersdorf AG, Hamburg, Germany), waterproofed the joint using silicon sealant and spray-painted the model matt black to imitate the absorbance and reflectance properties of a bank cormorant.

We attached these air and black bulb thermometers to a large cement breeze block as an anchor against wind and storms using iron bars (Fig. 1A). A square of rubber minimized heat transfer between the thermometer and bar. In this way, thermometers were held *ca* 50 cm above the ground. We placed the blocks as close as possible to each of the three colonies without disturbing the birds (between 20 and 80 m from nests), in locations that replicated the thermal properties of nest sites as closely as possible—e.g. positioned on raised rocks, with similar exposure to sun and prevailing winds. We programmed the iButtons to record temperature every 10 minutes and we manually downloaded the data every 1–3 weeks by communicating remotely with the iButtons via external cables connecting to the iButton holders (Fig. 1B). We took the mean from the two iButton recordings from each thermometer as *T*_air_ and *T*_bb_ for each 10-minute period.

### Wind speed and humidity measurements

Rates of heat transfer increase with wind speed. Under certain conditions, forced convection can have a greater influence on rates of heat dissipation than conduction and evaporation (Webb and King 1983). In warm environments, exposure to moderate wind speeds can help birds dissipate excess heat (Wolf and Walsberg, 1996). Humidity can also affect rates of heat transfer. Under more humid conditions, the rate at which birds can dissipate heat via evaporative water loss can be inhibited by as much as 36% (Gerson *et al.*, 2014).

To account for these effects in our statistical models, we obtained wind speed and relative humidity data for the study period from the South African Weather Service (SAWS) recording station closest to each colony and located on the coastline. Data for Jutten Island were obtained from the Cape Columbine station (32°49′S, 17°51′E), 30 km to the north of the colony; data for Robben Island were obtained from the Robben Island station (33°47′S, 18°22′E), 0.3 km from the colony; and data for the Stony Point colony were obtained from the Hermanus station (34°25′S, 19°13′E), 30 km east of the colony (Fig. S1). Wind speed (m s^−1^) and relative humidity (%) were recorded every hour over the study period.

### Focal thermoregulatory behaviours

We monitored adult bank cormorants at nests, focusing on behaviours considered to have a thermoregulatory function (hereafter thermoregulatory behaviour), e.g. postural adjustments and gular fluttering behaviour (see Table 1 for a list of focal behaviours). Postural adjustments allow birds to alter the amount of body surface exposed to air, changing the amount of body heat lost passively via radiation and convection (Cook and Leblanc, 2007; Ward *et al.*, 2008). For example, a cormorant increases its exposed body surface when it droops its wings or shifts from a sitting to an upright position, like crouching or standing. By so doing, it exposes its ventral parts (breast, belly and flanks), legs (thighs, tibias and tarsi) and feet (toes) to shed heat more effectively. Feet may serve as a particularly efficient organ for thermoregulation because cormorants can increase blood flow to their feet to such an extent that they serve a similar function to brood patches (Morgan *et al.*, 2003; Hart *et al.*, 2016).

**Table 1:** List of behaviours analysed and their presumed thermoregulatory function.

***Behaviour***	***Thermoregulatory function***
Sitting	Heat conservation
Crouching	Heat dissipation
Standing	Heat dissipation
Wings closed	Heat conservation
Wings drooped	Heat dissipation
Wings spread	Heat dissipation
Head tucked	Heat conservation
Head down	Heat conservation
Head up	Heat dissipation
Beak closed	Heat conservation
Beak open	Heat dissipation
Gular fluttering	Heat dissipation
Nest maintenance	None
Chick feeding	None
Egg rolling	None
Grooming	None
Bowing display	None
Aggressive display	None
Interaction with other birds	None
Nest shift with partner	None

Passive heat loss is only effective when body core temperature (*ca* 40°C; Wilson and Grémillet; 1996) exceeds *T*_air_. Once *T*_air_ approaches or equals body temperature, birds have to resort to evaporative heat loss to shed heat (Dawson, 1982). Respiratory cooling is one of the most common routes for evaporative heat loss, enabling cooling from moist surfaces of the mouth, pharynx and throat. Most bird species pant, but some groups, including cormorants, use gular fluttering instead (Bartholomew *et al.*, 1968). During gular fluttering the mouth is opened, the glottis and hyoid are depressed and the hyoid is flared, creating an open chamber where the tidal air from breathing passes over the surfaces, while the rapid, repetitive fluttering of the hyoid brings additional external air flow. The rate of blood flow to the buccal area is also elevated, increasing the amount of heat lost to evaporation (Lasiewski and Snyder, 1969). Because the muscles driving the hyoid during gular fluttering are small compared to the metabolically active mass of a bird, gular fluttering is considered to require low amounts of energy compared to panting, which produces significant amounts of metabolic heat (Lasiewski and Snyder, 1969). However, both panting and gular fluttering incur important costs in terms of water loss when there are prolonged, ultimately leading to dehydration (Smit and McKechnie, 2015). Furthermore, both routes of respiratory cooling, when sustained, also carry the risk of alkalosis, which is a dangerous build-up of blood CO_2_ (Smit *et al.*, 2016).

### Recording thermoregulatory behaviours

To estimate the occurrence of thermoregulatory behaviours and their duration, we set up video cameras (Sony DCR-SX22E) to film nesting birds from 20 to 80 m away from their nests. The cameras were mounted on tripods and protected by closed-circuit television (CCTV) housings. They were connected to digital video recorders designed for CCTV surveillance and powered via the main electrical grid (Robben Island) or via two deep-cycle 12 V batteries and an AC/DC power inverter (other two colonies). Footage was stored on an external hard drive. Depending on the distance to the colony, each camera filmed 1–5 nests. In order to increase the sample size, we changed the focal nests regularly (1–2 times a day) by adjusting the camera angle. The sex of filmed birds was unknown; male bank cormorants are only slightly larger than females and the sexes are not distinguishable in the field. We observed no nest desertion or predation while installing or retrieving cameras or while downloading data from temperature recorders.

### Analysis of thermoregulatory behaviour data

We only analysed footage collected between 10:00 and 16:00, as the quality of footage outside these times was affected by reduced light and glare from low sun angles. We randomly selected 41 hours of recordings out of a total of 268 hours, checked that these samples adequately reflected the range of temperatures to which the birds were exposed and analysed each hour in six 10-minute sessions, yielding *N* = 246 (6 × 41) samples for further analysis. We analysed the videos on a computer screen using JWatcher v1.0 (Blumstein et al. 2006), which allowed the observer to record behaviours in real time by assigning keyboard key codes to a list of 20 behaviours. Some behaviours were defined as being mutually exclusive, while others could occur simultaneously. A single observer watched all the videos, recording the start and end of each behaviour by pressing the designated key.

The duration of a focal thermoregulatory behaviour was expressed as a proportion of the time available in which it could occur in each 10-min (600 second) period. This was necessary because of the occurrence of mutually exclusive behaviours. For example, ‘gular fluttering’ could not occur during non-thermoregulatory behaviours like ‘nest maintenance’ or during heat conservation behaviours, such as when birds had their ‘head tucked’ (Table 1). Hence, the proportion of time during which each focal behaviour was undertaken (*P*) was calculated as follows:

$$ P=\frac{F}{600-\sum (E)}, $$

where $F$ is the duration (in seconds) of the focal behaviour and $E$ is the duration (in seconds) of all excluding behaviours.

### Modelling the relationship between temperature and gular fluttering

We modelled the relationships between both *T*_air_ and *T*_bb_ and the proportion of time birds spent gular fluttering. We considered gular fluttering to be the key focal thermoregulatory behaviour due to its central role in shedding heat efficiently in cormorants and its implications for water balance (Dawson 1982). Postural adjustments may influence thermoregulatory capacities, so the posture of the bird (two postures: sitting versus crouching or standing) was considered as a covariate of temperature. The position of wings relative to breast, belly and flanks may also influence bird thermoregulation, but this behaviour was not included because wing drooping was positively correlated with crouching (Table S1). Finally, wind speed and relative humidity were included as covariates in *T*_air_ models, because they may respectively increase and decrease heat loss in birds.

**Table 2:** Candidate models and model selection results for inflated beta regression modelling relating bank cormorant gular fluttering behaviour to black bulb temperature (*T*_bb_) and air temperature (*T*_air_)

*Type*	*Model*	*Number*	*pD*	*DIC*	*Deviance*	*∆DIC*
T_bb_	T_bb_ × Posture + NestID	O1	32.8	2875.39	2842.59	0.00
	T_bb_ + NestID	O2	29.0	2917.22	2888.91	42.56
	Intercept Only + NestID	O3	26.3	3010.05	2983.68	134.66
T_air_	T_air_ × Posture + NestID	A4	32.7	2916.85	2884.16	0.00
	T_air_ × Posture + Wind + NestID	A3	33.3	2917.34	2884.04	0.49
	T_air_ × Posture + Humidity + NestID	A2	37.2	2923.51	2886.33	6.66
	T_air_ × Posture + Humidity + Wind + NestID	A1	40.3	2927.04	2886.70	10.19
	T_air_ + NestID	A5	29.4	2952.71	2923.33	35.87
	T_air_ + Wind + NestID	A8	30.4	2954.32	2923.91	37.47
	T_air_ + Humidity + NestID	A7	33.8	2960.21	2926.36	43.36
	T_air_ + Humidity + Wind + NestID	A6	35.6	2962.49	2926.86	45.64
	Intercept Only + NestID	A9	26.4	3010.07	2983.65	93.22

Notes: pD = effective number of model parameters; DIC = deviance information criterion; ∆DIC = difference in DIC to the best supported model; Posture = a fixed effect with two levels denoting bird posture (sitting versus crouching or standing); Humidity = relative humidity; Wind = wind speed; NestID = random intercepts denoting the nest identify from which observation was drawn, present in all models. Models ranked by ∆DIC.

Because the response data were proportions of time spent gular fluttering, they could take any continuous value in the interval [0, 1]. Thus, we specified an inflated beta regression model with a mixed continuous–discrete distribution, a beta distribution to describe the continuous component and a Bernoulli distribution to give non-negative probabilities to the data points taking values = 0 or 1 (Ospina and Ferrari, 2010; 2012). These models are increasingly being used to model proportional responses in ecological studies (Richards, 2008; Harrison, 2015; Zuur *et al.*, 2015). The proportion of time gular fluttering (${p}_i$) was modelled as a linear function of the explanatory variables using a Beta-binomial distribution and a logit link function as follows:

$$ {y}_i\sim Bin\left({p}_{i,}{n}_i\right), $$

$$ {p}_i\sim Beta\left({a}_i,{b}_i\right) $$

$$ {a}_i=\theta \times{\pi}_i, $$

$$ {b}_i=\theta \times \left(1-{\pi}_i\right), $$

$$ logit\left({\pi}_i\right)={\beta}_1+{\beta}_2{x}_i+{z}_{i,j} $$

$$ {z}_i\sim N\left(0,{\sigma}_j^2\right), $$

where ${y}_i$ are individual gular fluttering observations, ${n}_i$ is the number of seconds of each observation (trials), $\theta$ is the dispersion parameter (estimated by the model), the *β*s are the coefficients to be estimated for any fixed-effects, ${z}_{i,j}$ denotes a random effect accounting for repeat observations (*i*) within nests (*j*) and ${x}_i$ is a covariate vector.

We modelled the effect of *T*_bb_ and *T*_air_ separately. For *T*_air_ the maximal model included an interaction between temperature and the bird’s posture (sitting versus crouching or standing), wind speed and relative humidity. We omitted wind speed and relative humidity for *T*_bb,_ as this measurement should already incorporate the effect of these variables (see above). We implemented our models using Monte-Carlo Markov Chain (MCMC) estimation in JAGS (v.4.1.0; Plummer, 2003) via the ‘jagsUI’ library (v. 1.4.2; Kellner, 2016) for programme R v.3.2.3. The uninformative priors were $N(0,{10}^{-3})$ for the coefficients of the linear models (*β*) and $N\left(0,1/{\sigma}^2\right)$ for the hyper-priors associated with the random effects (*z*), where the precision was $1/{\sigma}^2\sim Gamma\left(0.001,0.001\right)$. Inference was based on three chains of 120 000 samples, with the first 20 000 discarded as burn-in, and thinned to every fifth observation to increase the effective MCMC sample size for the same amount of computer memory. We undertook model simplification (see Table 2 for all candidate models) using the deviance information criterion (DIC), examined the posterior distributions of parameters dropped from the models and presented means ±95% credible intervals unless otherwise specified. Finally, we assessed model fit (Fig. S2 and S3) and convergence both visually and using Gelman–Rubin diagnostics (Brooks and Gelman, 1998); all $\hat{R}$ values ≤1.02.

### Predicting the effect of climate change on thermoregulation behaviour

To predict the effect of climate change on gular fluttering behaviour in breeding bank cormorants, we focused on sitting birds, because they should be more sensitive to heat (i.e. start gular fluttering at lower temperatures) than crouching or standing birds. After modelling the *T*_air_ at which sitting cormorants gular flutter for 50% of the time on average (see above), we used this temperature threshold to predict how long sitting birds should gular flutter in the year 2100. We used hourly *T*_air_ recordings from SAWS stations between April and August 2012 as a baseline (*N* = 11 015; Fig. S1). To this baseline, we added the forecasted increase in average temperature for the southern African region following two representative concentration pathway (RCP) scenarios (IPCC 2014). These different pathways describe two possible climate futures depending on the levels of greenhouse gases emitted in the coming years. RCP 2.6 involves radical human mitigation to limit the increase in temperature (Van Vuuren *et al.*, 2011), whereas RCP 8.5 represents a situation of high human population in the absence of climate change policies (Riahi *et al.*, 2011). By 2100, RCP 2.6 and RCP 8.5 predict an increase in mean surface temperature in southern Africa during the bank cormorant breeding season of, respectively, 1.1°C and 5.4°C relative to 1986–2005 (IPCC 2013), period to which we associate the temperature data collected in this study, for reasons of convenience. This represents an increase of *ca* 1.7°C and 6°C relative to the pre-industrial baseline (IPCC 2014).

## Results

A total of *N* = 33 050 *T*_bb_ and *T*_air_ measurements (Fig. S4) and *N* = 10 989 wind speed and relative humidity measurements (Fig. S5) were recorded over the study period. *T*_bb_ was higher than *T*_air_ during the daytime, but not during the night (Fig. 1C–E and S6). Overall, *T*_air_ was strongly correlated with *T*_bb_ over the entire study period (Spearman rank correlation *R* = 0.93, Fig. S7).

For both *T*_bb_ and *T*_air_, the best fitting models (O4 and A1, Table 2) contained interactions between temperature and bird posture as the only explanatory variables. A model containing wind speed (A3, Table 2) was within a ∆DIC ≤ 2 of the best supported models for *T*_air_, but the 95% credible interval estimates for the wind effect included zero in this model. Thus, while wind speed might influence gular fluttering behaviour, we did not consider this effect further based on model parsimony. *T*_bb_ provided a more parsimonious explanation of the variation in proportion of gular fluttering than *T*_air_ (∆DIC was 41.57 in favour of the T_bb_ model).

**Figure 2:**
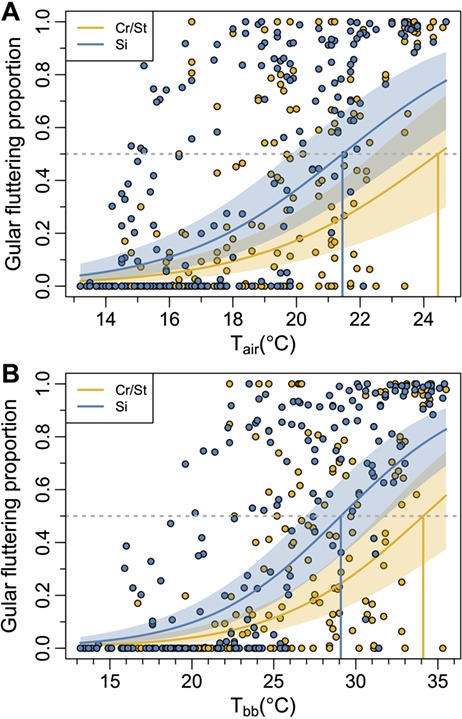
Modelled relationships between temperature and the proportion of time bank cormorants spent gular fluttering. Models are presented for (A) air (*T*_air_) and (B) black bulb (*T*_bb_) temperatures. Observed data (points), the predicted mean (solid lines) and 95% credible intervals (shaded areas) from the beta-binomial regression for birds that were sitting (Si, blue) versus those with an upright posture (crouching or standing: Cr/St, yellow) are shown for both temperatures. The coloured lines that intersect the x-axis mark the mean predicted temperature at which birds would have to spend 50% of the time gular fluttering (denoted by the dotted grey line).

The time birds spent gular fluttering increased with both *T*_air_ and *T*_bb_ but did so faster in sitting birds than in birds that were crouching or standing (Fig. 2). Based on the best-fitting models, the gular fluttering proportion was predicted to exceed 0.5 (i.e. birds gular fluttering for >50% of time on average) in sitting bank cormorants after *T*_air_ = 21.4°C (95% CI for gular fluttering proportion, 0.33–0.66) or *T*_bb_ = 29.1°C (0.35–0.63; Fig. 2). For crouching or standing birds, the corresponding temperatures were *T*_air_ = 24.4°C (0.29–0.70) and *T*_bb_ = 34.1°C (0.32–0.67; Fig. 2).

**Figure 3:**
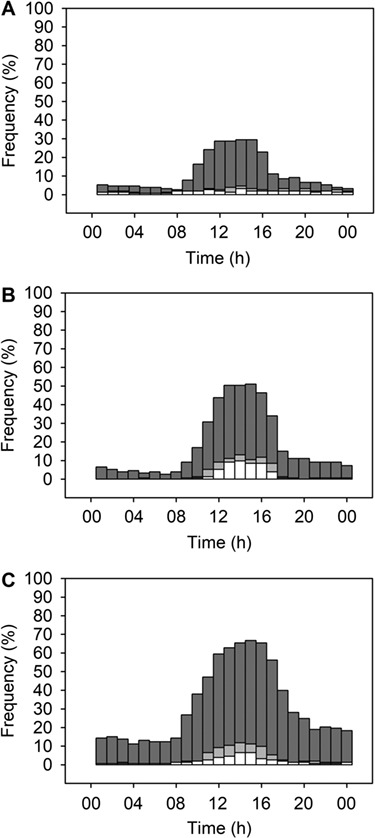
Frequency of hourly air temperatures (*T*_air_) ≥21.4°C according to time of day during the bank cormorant breeding season (April–August). Situations are presented for (A) Jutten Island, (B) Robben Island and (C) Stony Point colonies. The 21.4°C threshold represents the *T*_air_ above which sitting (the posture where birds are the most sensitive to heat) cormorants gular flutter for more than 50% of the time on average (Fig. 2). The proportion of time for which *T*_air_ ≥ 21.4°C is given for the study year 2012 (white bars, *N* = 11 015) and projected for the year 2100 following the RCP 2.6 (grey bars) or RCP 8.5 (dark grey bars) scenarios. RCP 2.6 and RCP 8.5 refer to IPCC (2013) scenarios predicting increases in global average surface temperature of respectively ca. 1.1°C and 5.4°C relative to the 2012 study period.

The proportion of breeding bank cormorants gular fluttering for at least 50% of the time is forecast to increase in proportion to the expected increase in *T*_air_ (Fig. 3). In 2012, *T*_air_ was ≥21.4°C during 1.9% of the day (range 1.6–2.0% for the 3 colonies). This is projected to increase to 3.0% (RCP 2.6) or 20.5% (RCP 8.5) of the day in 2100. During the hottest hours of the day (*ca* 11:00–17:00), the occurrence of *T*_air_ ≥ 21.4°C is expected to reach up to 70% by 2100 following the RCP 8.5 scenario (Fig. 3C).

## Discussion

Our study shows that videography is a relevant technique for non-invasive studies of thermoregulatory responses in wild and threatened seabirds sensitive to human disturbance. Continuous video recording provided detailed footage of individuals exposed to a wide range of environmental conditions. Our results allow us to predict how breeding bank cormorants might adjust their thermoregulatory behaviour to future climate change. We encourage the use of digital photography and videography for the remote study of thermoregulatory behaviour. This approach is particularly well adapted to the study of breeding birds as filming is facilitated when birds are nest bound, but studying wild mammals or even ectothermic species might be possible under certain circumstances.

Our observations suggest that breeding bank cormorants use a suite of behavioural adjustments to help manage heat budgets during hot weather events. These behavioural adjustments are likely to become increasingly important with rising temperatures, with breeding bank cormorants predicted to spend considerably more time engaging in behaviours that promote heat dissipation under the two climate change scenarios examined. By exploring the relationship between thermoregulatory behaviours, we were able to identify potential trade-offs that bank cormorants face during periods of heat stress, with likely implications for reproductive success and the persistence of populations.

### The relative influence of climatic variables on behaviour

Studies exploring the impact of temperature on animal behaviour often focus on the effect of *T*_air_. However, the ‘thermal load’ (i.e. the balance of heat gain and heat loss between an animal and its environment) depends on a variety of different environmental parameters in addition to *T*_air_ (Angilletta, 2009). Bank cormorants, like many seabirds, nest in exposed locations with little protection from solar radiation and the wind. In addition to recording *T*_air_, we also measured *T*_bb_, which integrates the combined influence of the radiative and convective environment (i.e. the sun and wind).

We found that *T*_air_ was strongly correlated with *T*_bb_ (Fig. S7), but *T*_bb_ was significantly greater than *T*_air_ by up to 15°C (Fig. S7). Breeding bank cormorants were mostly exposed to *T*_bb_ values ~15°C, but these occasionally rose to ~40°C (Fig. S4B), contrasting markedly with the cold ocean temperatures experienced during foraging (typically ≤15°C; Demarcq *et al.*, 2003). *T*_bb_ better explained the variation in the time spent gular fluttering than *T*_air_ (Table 2), suggesting that solar radiation and wind are important drivers of this behaviour. However, behavioural responses varied with *T*_air_ in a similar manner to *T*_bb_ (Fig. 2B), suggesting that *T*_air_, which can be more easily measured and is modelled in climate change scenarios, can be confidently used to predict responses to increasing global temperatures.

Humidity also influences the potential for evaporative heat loss (Webb and King, 1983; Gerson *et al.*, 2014), but our most parsimonious models for *T*_air_ did not include this variable (Table 2), suggesting a limited effect of humidity over the range to which birds were exposed during this study (*ca* 50–90%; Fig. S5C). Future studies should focus on examining bird behaviour at high temperatures, where the influence of other variables might be more significant.

### Thermoregulation and trade-offs with parental behaviour

Gular fluttering is used by several bird groups to help dissipate excess heat through enhanced evaporative heat loss (Bartholomew *et al.*, 1968). Although it is efficient at rapidly dissipating heat, it incurs high costs in terms of water loss (e.g. Lasiewski and Snyder, 1969; Hochscheid *et al.*, 2002; Smit and McKechnie, 2015). Even at relatively low temperatures, well within conditions currently experienced by breeding bank cormorants, parents attending nests spend a considerable proportion of their time gular fluttering (Fig. 2). This relationship is strongly modified by posture, such that bank cormorants can decrease the proportion of time gular fluttering by crouching or standing (compared with sitting), by 30% at 22°C (Fig. 2A). Standing and crouching increases the skin surface exposed to air, including from legs and feet, promoting heat dissipation through convective processes and radiation.

Thus, postural adjustments use passive forms of heat transfer, which are less costly in terms of energy and water demand than gular fluttering, but may incur thermoregulatory costs to eggs or small chicks. Indeed, breeding cormorants must not only manage their own heat, energy and water budgets but also the demands of their offspring. Eggs and small chicks are unable to generate sufficient metabolic heat to maintain egg or body temperatures and by adjusting to an upright posture, the nest contents may be exposed to cooler temperatures leading to hypothermia. In addition, when adults crouch or stand, they increase the risk of nest depredation by aerial predators (Orta, 1992) such as kelp gulls *Larus dominicanus* (Hockey *et al.*, 2005)*.* Once chicks approach thermal independence (from 3-weeks old), adults can finally crouch over their brood, thereby expose their feet and increase their body surface for passive cooling. Standing can only be adopted when chicks become large enough to discourage predators (from 5-weeks old). By then, chicks are so large that the nest cannot accommodate both the brood and the rearing parent, which usually stands by the nest. Birds are therefore constrained by their breeding stage and have to use evaporative cooling at relatively low *T*_air_, irrespective of body posture, during the incubation and small chick-rearing stages.

### Thermal adaptations in diving seabirds

Diving seabirds such as bank cormorants face a particularly acute thermoregulatory challenge because they experience a wide range of environmental conditions. In addition to avoiding hyperthermia while breeding, they must also minimize heat loss while diving in ≤15°C waters. The predominantly black plumage of cormorants may assist with rapid heat gain following dives (Cook *et al.*, 2012) but may mean cormorants face greater heat loads from solar radiation during nest attendance. The ‘partially wettable’ plumage of cormorants (Grémillet *et al.*, 2005) traps enough air to create an insulating layer, thereby limiting heat loss to the aquatic environment. In addition, diving cormorants can drop their core body temperature by as much as 12°C (Bevan *et al.*, 1997) to reduce the metabolic costs of diving for prey (Wilson and Grémillet, 1996). This hypothermic capacity may also assist cormorants in managing heat stress to some extent, enabling them to spend longer at their nests before body temperatures reach lethal levels (average foraging trip duration over the breeding period, 106 min; Botha, 2014).

### Implications of climate change for bank cormorants

For bank cormorants, breeding may become a challenging activity in the near future. The most extreme climate projection (RCP 8.5) suggests that, by 2100, situations when nest-bound birds are gular fluttering for over 50% of the time will occur with a probability of up to 70% during the warmest hours of the day (11:00–17:00) (Fig. 3C). In Cape gannets *Morus capensis*, evaporative water loss amounts to 100% of daily ingested water after 7.5 hours of gular fluttering (Hochscheid *et al.*, 2002). Hence, prolonged gular fluttering may lead to dehydration, forcing bank cormorants to leave the nest before the return of their partner from the sea. Bank cormorants in Namibia have already been observed abandoning chicks during high temperature events (Sherley *et al.*, 2012), and heat waves are expected to increase in frequency and intensity in southern Africa (Kruger and Sekele, 2013). Future studies should attempt to estimate the rate of water loss in bank cormorants during evaporative cooling and its consequences on individual fitness and demography.

The recent decrease in bank cormorant numbers is believed to be largely due to competition with industrial fisheries for west coast rock lobsters *Jasus lalandii* (Sherley *et al.*, 2017). However, climate change may represent an additional threat to this endangered seabird. Bank cormorants are winter breeders in South Africa, but it is not clear what factors influence their phenology, as there is no evidence that prey availability is higher in winter. The benefits derived from breeding in cooler conditions (with less heat stress) might outweigh the risk of nest failures caused by storm activity (nests washed away by extreme waves) in winter (Sherley *et al.*, 2012). The forecasted increase in *T*_air_ and in extreme wave heights (Theron *et al.*, 2010) may result in reduced breeding success. Unfortunately, bank cormorants have limited options to adapt either their range (cf. Chen *et al.*, 2011) or their breeding phenology (cf. Ovaskainen *et al.*, 2013) because they already breed in winter and are located at the southern tip of the African continent.

The southern Benguela has undergone an ecosystem change that is believed to result from a combination of fishing pressure and environmental forcing (Blamey *et al.*, 2015). Within this context, west coast rock lobster populations have shifted the core of their distribution from the west coast to the south-west coast of South Africa since *ca* 1990, moving away from several traditional bank cormorant breeding grounds and into waters that are naturally devoid of adequate seabird breeding habitat (islands). Scarcer food resources may further exacerbate thermoregulatory challenges, requiring longer foraging trips by breeding cormorants (Piatt *et al.*, 2007), resulting in partners needing to attend the nest for longer periods and reducing the rate at which energy and water can be obtained. At the same time, increasing temperatures may increase the requirements for these resources in both adults and chicks and expose nests to greater risk of predation. The consequences of changes in prey availability and increases in temperature are difficult to predict but may act in concert to place additional pressures on already endangered seabird populations.

## Supplementary Material

Cook_et_al_CONS_PHYSIOL_HeatStress_SUPPLEMENT_R2_coz109
