# Supplementary material for: Parenting in a warming world: thermoregulatory responses to heat stress in an endangered seabird
Source: Conserv Physiol. 2020 Jan 18;8(1):coz109. doi: 10.1093/conphys/coz109 (PMC6970236; doi:10.1093/conphys/coz109)
Supplement: Cook_et_al_CONS_PHYSIOL_HeatStress_SUPPLEMENT_R2_coz109 [file cook_et_al_cons_physiol_heatstress_supplement_r2_coz109.pdf]

Parenting in a warming world: thermoregulatory responses to heat stress in an endangered seabird

Timothée R. Cook[1,2,*], Rowan Martin[1], Jennifer Roberts[1], Henry Häkkinen[3], Philna Botha[1,4], Corlia Meyer[5], Emilee Sparks[1], Leslie G. Underhill[4], Peter G. Ryan[1], Richard B. Sherley[3,6]

[1]FitzPatrick Institute of African Ornithology, DST-NRF Centre of Excellence, University of Cape Town, Rondebosch, 7701, South Africa
[2]BLOOM Association, 62 bis avenue Parmentier, 75011 Paris
[3]Centre for Ecology and Conservation, University of Exeter, Penryn, TR10 9FE, United Kingdom
[4]Animal Demography Unit, Department of Biological Sciences, University of Cape Town, Rondebosch, 7701, South Africa
[5]Centre for Research on Evaluation, Science and Technology, Stellenbosch University, Stellenbosch, 7600, South Africa
[6]Environment and Sustainability Institute, University of Exeter, Penryn, TR10 9FE, United Kingdom

***Email: timothee.cook@gmail.com

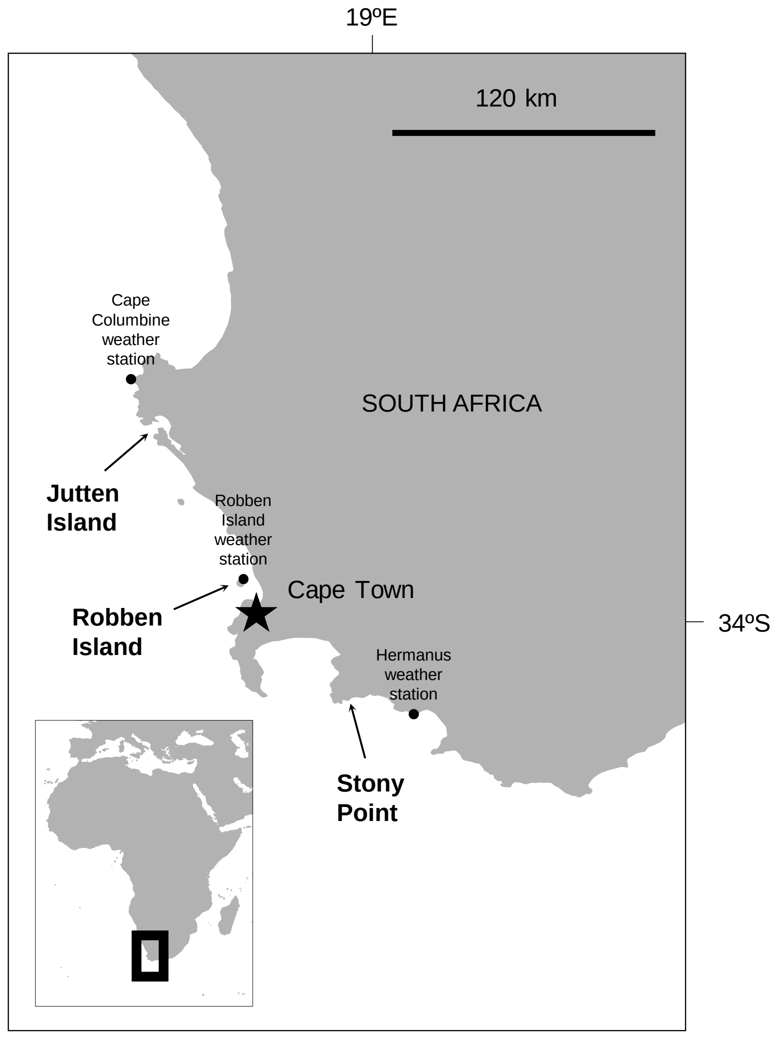

Figure S1: General map of the study area. Location of the three study colonies and corresponding South African Weather Service stations are shown. Weather stations recorded T_{air}, humidity and wind speed every hour over the entire breeding season (April–August, N=11,015 in total).

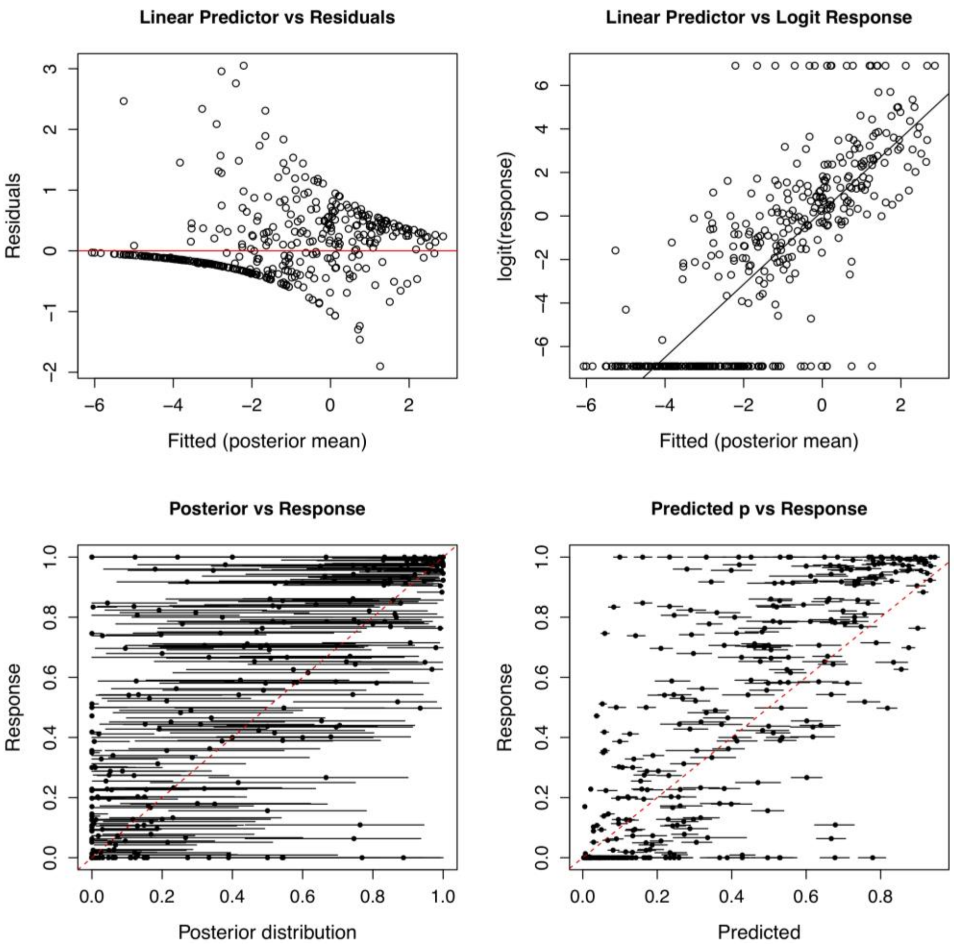

Figure S2: Model fit diagnostics for T_{bb} model O1 (Table 2). Top left: Fitted values (linear predictor) versus Pearson's residuals. Top right: fitted values (posterior mean of the linear predictor) versus logit response. Bottom left: posterior mean (point) and 95% distribution (horizontal lines) versus observed response. Bottom right: posterior predicted p-value versus observed response.

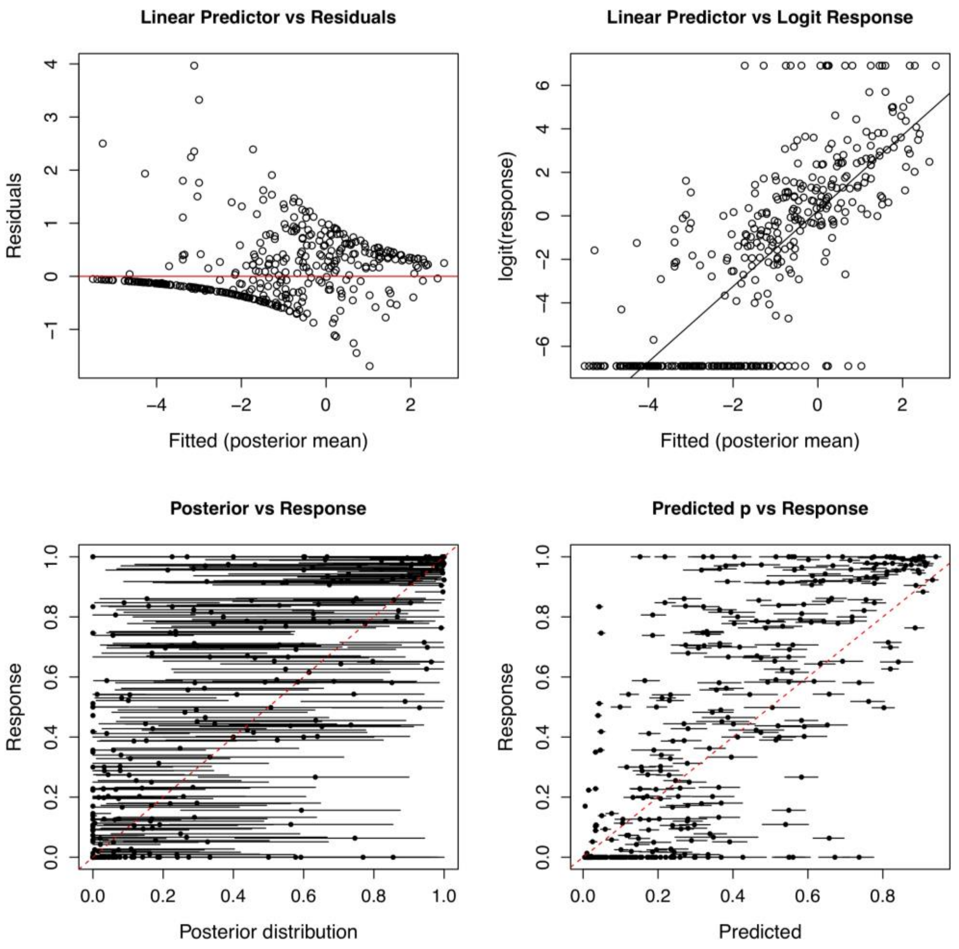

Figure S3: Model fit diagnostics for T_{air} model A4 (Table 2). Top left: Fitted values (linear predictor) versus Pearson's residuals. Top right: fitted values (posterior mean of the linear predictor) versus logit response. Bottom left: posterior mean (point) and 95% distribution (horizontal lines) versus observed response. Bottom right: posterior predicted p-value versus observed response.

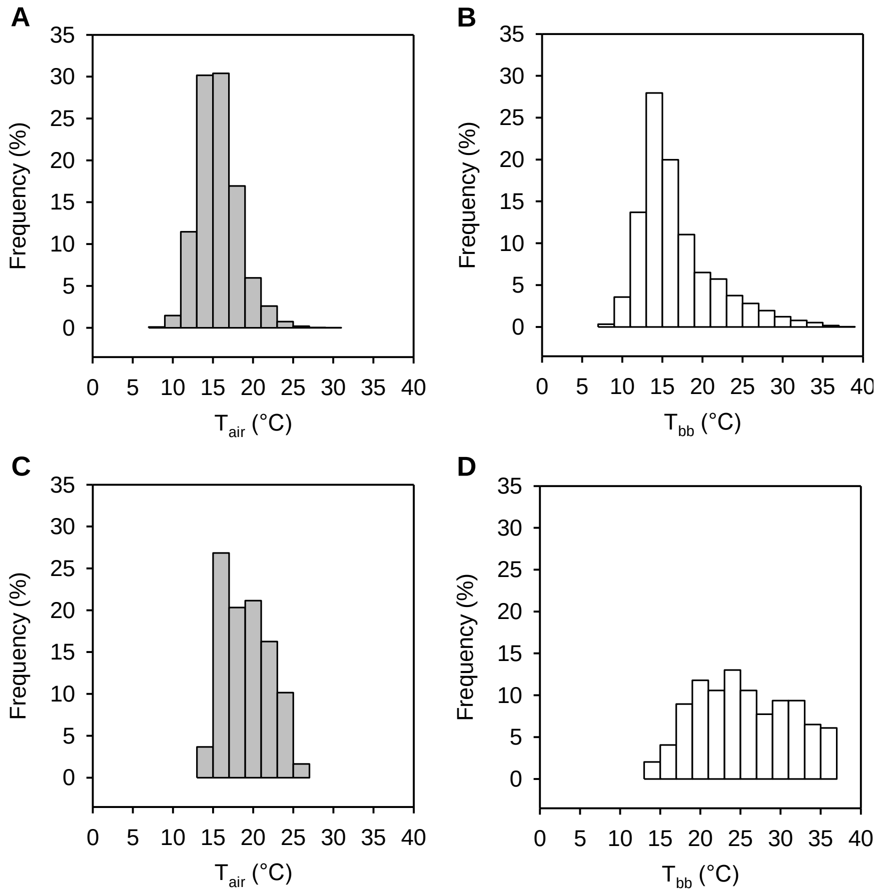

Figure S4: Temperatures recorded at study colonies on Jutten Island, Robben Island and Stony Point, April–August 2012. Frequency distribution of (A) T_{bb} and (B) T_{air} recorded over the study period (*N*=33,050). Frequency distribution of (C) T_{bb} and (D) T_{air} recorded concurrently to the sampled video footage for behaviour analysis (*N*=246).

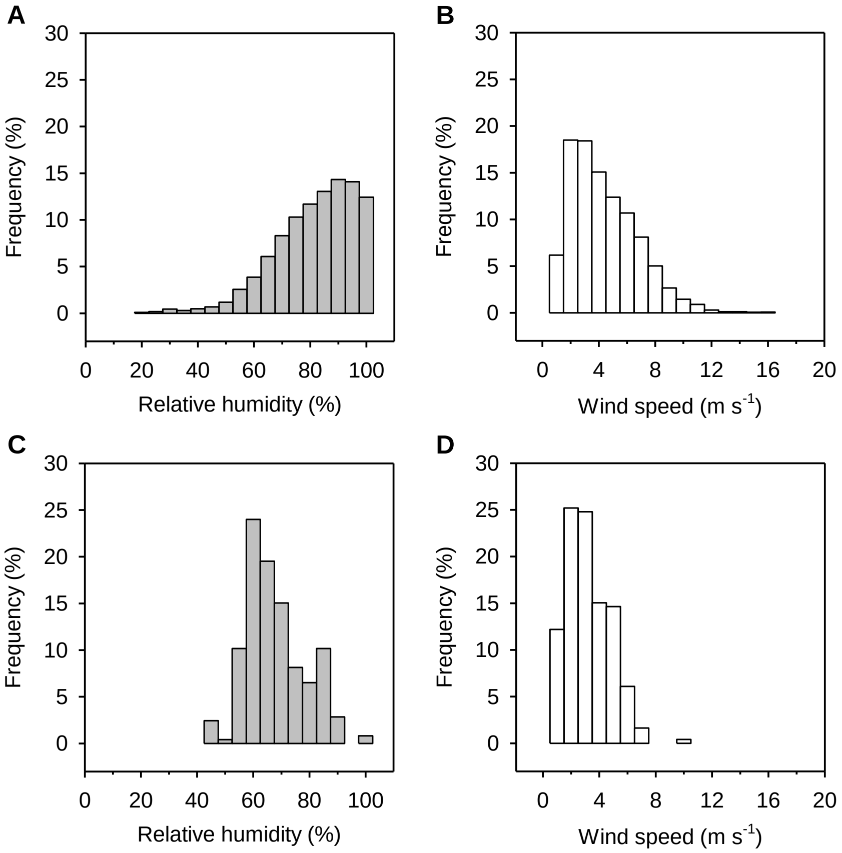

Figure S5: Relative humidity and wind speed recorded at the study colonies of Jutten Island, Robben Island and Stony Point, April–August 2012. Frequency distribution of (A) relative humidity (*N*=10,989) and (B) wind speed (*N*=10,692) recorded over the study period. Frequency distribution of (C) relative humidity and (D) wind speed recorded concurrently to the sampled video footage for behaviour analysis (*N*=246).

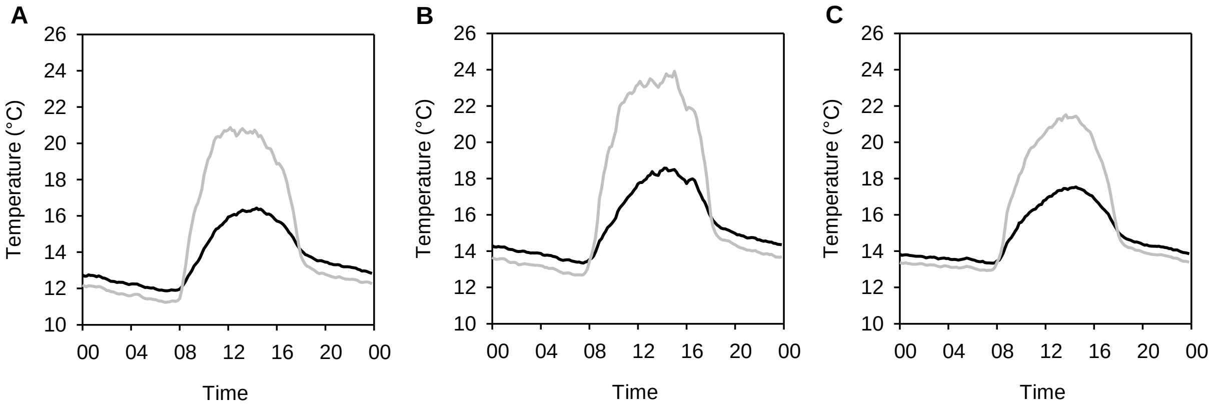

Figure S6: Relationship between temperature and time of day. Mean T_{air} (black line) and T_{bb} (grey line) recorded at the study colonies of (A) Jutten Island (*N*=8,819), (B) Robben Island (*N*=6,127) and (C) Stony Point (*N*=18,104) between April and August 2012.

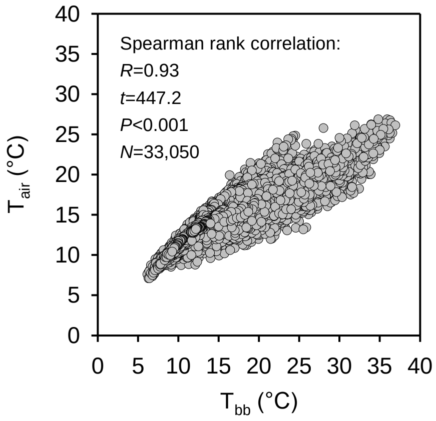

Figure S7: Relationship between T_{air} and T_{bb}. Temperatures were recorded at the study colonies of Jutten Island, Robben Island and Stony Point between April and August 2012.

Table S1: Correlation matrix giving Spearman's rank correlation coefficients for thermoregulatory behaviours of nesting bank cormorants. Bold indicates statistical significance ($p < 0.05$). Italics indicate mutually exclusive behaviours. Behaviour names are abbreviated: Si = sitting, Cr = crouching, St = standing, WC = wings closed, WP = wings propped, WS = wings spread, HT = head tucked, HD = head down, HU = head up, BC = beak closed, BO = beak open, GF = gular fluttering.

	Si											
Cr	*-0.62*	Cr										
St	*-0.66*	*-0.17*	St									
WC	0.07	-0.31	0.22	WC								
WP	-0.12	0.29	-0.12	*-0.38*	WP							
WS	0.04	-0.04	-0.02	-0.01	*-0.03*	WS						
HT	0.03	*-0.10*	*0.06*	0.20	*-0.10*	*-0.02*	HT					
HD	0.23	-0.09	-0.21	0.00	0.12	-0.06	*-0.09*	HD				
HU	0.02	0.10	-0.12	-0.19	-0.12	0.09	*-0.38*	*-0.67*	HU			
BC	0.03	-0.19	0.15	0.27	0.04	-0.06	0.33	0.56	-0.82	BC		
BO	-0.02	0.19	-0.15	-0.27	-0.04	0.06	*-0.33*	-0.55	0.82	*-1.0*	BO	
GF	0.05	0.13	-0.19	-0.24	-0.13	0.08	*-0.34*	-0.60	0.92	*-0.9*	0.9	GF
